# Co-Design of a Disease Activity Based Self-Management Approach for Patients with Rheumatoid Arthritis

**DOI:** 10.31138/mjr.32.1.21

**Published:** 2021-03-31

**Authors:** Marieke J. Spijk-de Jonge, Sofie H. M. Manders, Anita M. P. Huis, Glyn Elwyn, Mart A. F. J. van de Laar, Piet L. C. M. van Riel, Marlies E. J. L. Hulscher

**Affiliations:** 1 Radboud University Medical Centre, Radboud Institute for Health Sciences, IQ Healthcare, Nijmegen, The Netherlands; 2 Venray, The Netherlands; 3 The Dartmouth Institute for Health Policy and Clinical Practice, Lebanon (NH), United States of America; 4 University of Twente, Department of Psychology, Health and Technology, Enschede, The Netherlands; 5 Bernhoven, Department of Rheumatology, Uden, The Netherlands

**Keywords:** DAS28, patient education, patient empowerment, rheumatoid arthritis, tight control, treat-to-target

## Abstract

**Objective:**

The systematic development of an intervention to improve disease activity-based management of rheumatoid arthritis (RA) in daily clinical practice that is based on patient-level barriers.

**Methods:**

The self-management strategy was developed through a step-wise approach, in a process of co-design with all stakeholders and by addressing patient level barriers to RA management based on disease activity.

**Results:**

The resulting DAS-pass strategy consists of decision supportive information and guidance by a specialised rheumatology nurse. It aims to increase patients’ knowledge on DAS28, to empower patients to be involved in disease management, and to improve patients’ medication beliefs. The decision supportive information includes an informational leaflet and a patient held record. The nurse individualises the information, stimulates patients to communicate about disease activity, and offers the opportunity for questions or additional support.

**Conclusion:**

The DAS-pass strategy was found helpful by stakeholders. It can be used to improve RA daily clinical practice. Our systematic approach can be used to improve patient knowledge and self-management on other RA related topics. Also, it can be used to improve the management of other chronic conditions. We therefore provide a detailed description of our methodology to assist those interested in developing an evidence-based strategy for educating and empowering patients.

## INTRODUCTION

International guidelines recommend rheumatologists to manage Rheumatoid Arthritis (RA) based on disease activity.^1,2^ This implies that disease activity should be monitored regularly, and medication should be adapted accordingly.^3^ The preferred treatment strategies tight control and Treat-to-Target are based on this principle. DAS28 is a validated and frequently used disease activity measure in RA. The DAS28 provides a value between 0 and 10, indicating level of disease activity. A DAS28 value equal to or below 3.2 indicates low disease activity and a value above 3.2 indicates moderate or high disease activity.

Although tight control/Treat-to-Target has internationally become part of ‘good clinical practice’, there is room for improvement. Notwithstanding scientific publications, meetings, organizational changes and time-saving interventions to implement it in daily practice, RA management based on disease activity is suboptimal.^4–6^ Despite the profound benefits for treatment outcomes with respect to patient functionality, pain and joint damage, patients are unaware of the importance of keeping disease activity low.^7–13^ Several studies show that patients are generally unaware of the long term consequences of high disease activity.^5,14^

Improving patient involvement in RA care could help to further improve RA management based on disease activity. Research shows that patients’ lack of knowledge on (the importance of) disease activity, and their reservations towards changing medication regimen hinder RA management based on disease activity.^5,14–19^ Patients do not always want to change their medication regimen when their rheumatologist thinks it is necessary: medication changes are highly associated with the consequences of disease on their current health status (eg, pain, fatigue, and current functionality) as opposed to biomedical aspects of disease reflected by disease activity and consequently progression of joint damage.^15–18^ Moreover, patients’ fear of side effects and losing control over their disease makes them accept their current health state and prefer their current treatment.^16,19^ These patient preferences do not seem to be informed decisions, as patients lack knowledge about disease activity and its long-term consequences. In addition, patients with RA do wish to be more informed and involved.^5,14,20–23^

We systematically developed a self-management strategy to improve RA management based on disease activity in a process of co-design with all stakeholders and by addressing patient level barriers, focusing on patients’ knowledge and empowerment. In this article, we describe the development process of this strategy because this approach fits the current paradigm of shared decision making between physicians and patients. Care for chronic diseases is increasingly designed around the idea that patients should have a central role in their own care.^24^ There are several chronic diseases where patients’ expertise about their own lives can be utilised more to manage the great variation and heterogenic character of a disease.^25^ The DAS-pass strategy is an example of how generic, evidence-based information can be tailored for patients to develop an instrument that is feasible for use in daily clinical practice. Hence, we provide a detailed description of our methodology to assist those interested in improving patient education by developing an evidence-based strategy for empowering and educating patients with chronic diseases.

## MATERIALS AND METHODS

A stepwise approach was taken to develop a patient-centred strategy tailored to patient-level barriers to disease activity-based management of RA, which is an important success factor for implementing evidence into daily clinical practice.^26,27^ Furthermore, the strategy was based on successful strategies from other chronic diseases and it was systematically developed in cooperation with stakeholders. The development process consisted of preliminary work, a content phase and a lay out phase (**Figure 1**).

**Figure 1.**
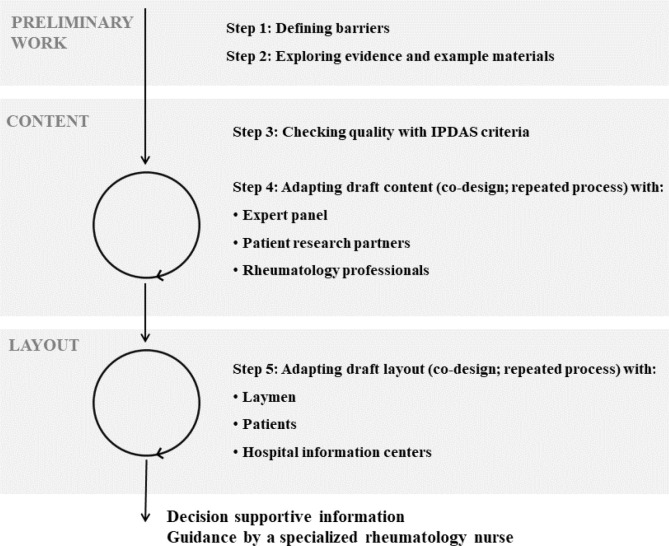
Stepwise development of the DAS-pass strategy (process map).

### Preliminary Work

The preliminary work in developing the strategy consisted of defining patient-level barriers and of exploring the evidence and example materials on targeting the identified barriers.

#### Step 1: Defining barriers

Within our research group, van Hulst et al. explored barriers to RA management based on disease activity by performing 28 semi-structured interviews (14 rheumatologists and 14 patients) and 5 focus groups (2 with rheumatologists and 3 with RA patients).^5,14^ They found that several of the barriers exist on the level of the patient. According to both patients and rheumatologists, patients lack knowledge on the important role of disease activity in RA. Patients who do mention to be familiar with disease activity doubt the relevancy of the outcome, or do not realise that high disease activity levels influence long-term functionality and joint damage. Other studies underwrite the finding that patients do not always want to adapt medication in case of active disease.^16,19^ In addition, while rheumatologists are interested in objective measures of disease status and the progression of damage, patients are more interested in the subjective aspects of the disease like pain, fatigue, and current functionality.^15,17–19^

In summary, our barrier analysis led to three patient-level barriers: 1) most patients are unfamiliar with disease activity measures; 2) many patients are not equipped to be involved in treatment (decisions); and 3) patients generally prefer not to change their current medication regimen. Based on these barriers, our strategy aims to improve patients’ knowledge on (the importance of) disease activity, empower patients to be more involved in their treatment (decisions), and improve patients’ beliefs about medication (changes).

#### Step 2: Exploring evidence and example materials

Based on previous research, we know that patient-centred strategies are effective in improving disease management.^27–29^ Decision aids and decision support interventions are designed to facilitate and improve patient-centredness. Randomised trials show that they stimulate patients to take a more central role in health-care and improve patients’ knowledge about treatment options and expectations. They also appear to lead to treatment decisions that better fit patients’ preferences and to improved communication between patients and physicians.^30,31^ Furthermore, we know that patients need knowledge as well as empowerment to be able to play an important role in their own healthcare.^32^

From diabetes mellitus research, we learned that a patient-held record can improve treatment outcomes (HbA1c and diastolic blood pressure).^28^ Furthermore, the involvement of a nurse is effective in educating patients, facilitating adherence to treatment and the self-efficacy of the patients, while also embedding the improvement strategy in daily clinical practice.^27,33,34^

Based on this evidence, our self-management approach aims to include decision supportive information (including patient held records) and nurse-led strategies designed to improve patient-centredness.

### Content phase

After the preliminary work, a coherent draft version of the content of the decision supportive information was developed as well as draft goals were formulated in a checklist to navigate nurses in leading the patient-centred strategy. During the content phase, the draft materials were improved through applying the International Patient Decision Aids Standards (IPDAS) criteria and in co-design with all stakeholders.

#### Step 3: Developing content while applying IPDAS criteria

The IPDAS collaboration has developed elaborate criteria for rating the quality of existing patient decision aids and to help designing high quality decision aids.^33^ Our decision supportive information has many commonalities with decision aids. Both aim to increase patients’ knowledge about (treatment of) their disease and to empower patients to communicate with their practitioners about it. Therefore many, though not all, of the IPDAS criteria were helpful in the development of the decision supportive information. The IPDAS criteria were used to improve the quality of the decision supportive information by checking the comprehensiveness, presentation, and wording of the content.

#### Step 4: Adapting draft content

The draft content of the information was finalised by consulting an expert panel, two patient research partners, and eight rheumatology professionals several times. After each consultation, the draft content of the information was adapted. This cycle continued until there were only minimal remarks from the expert panel, patient research partners and professionals. This procedure is demonstrated by the cycle in the process map (**Figure 1**).

The expert panel consisted of MH (expert on implementation strategies), GE (expert on shared decision making), and LvH (expert on barriers to RA management based on disease activity). They were asked to review the content of the decision supportive information from their own expertise. Two patient research partners, ie, RA patients who are trained to cooperate in scientific research, collaborated in the development of the decision supportive information. They know the principles of performing research and are experts on representing the patient point of view in (the treatment of) their disease. With help of the patient research partners, content of the decision supportive information was adapted in several meetings. To make sure that the decision supportive information represented daily clinical practice, four rheumatologists (including PvR) and three specialized nurses from different hospitals cooperated in the development process. They were asked to provide feedback on the content of the decision supportive information.

#### Layout phase

After the layout of the decision supportive information and nurse checklist were developed, the layout of the strategy was developed. By consulting laymen, hospital information centres, and patients several times, it was ensured that the information was presented clearly, and was supportive of its content.

#### Step 5: Adapting draft layout

At the beginning of the layout phase, three laymen were consulted. The laymen had no expertise or connection to RA. They were asked to give feedback on layout and wording of the draft information in a meeting with the researchers. The draft was adapted after feedback of the laymen. Then, the layout of the draft information was finalized by consulting five patients and two hospital information centres several times. This procedure is demonstrated by the cycle in the process map (**Figure 1**). After each consult, the draft layout of the information was adapted. This cycle continued until there were only minimal remarks from patients and hospital communications centres.

Five randomly selected RA patients were asked to review the draft. They were asked about their opinion on comprehensibility of information and on different layout options. The first time, the patients were consulted in a face-to-face meeting. Subsequently, they were consulted via e-mail several times.

The patient communication departments of two different hospitals provided feedback on the layout of the decision supportive information, based on their experience in informing patients about their disease and its treatment.

## RESULTS

Following a stepwise approach, we developed the DAS-pass self-management strategy based on identified barriers, on previous research, and in a process of co-design with all stakeholders. In summary, our barrier analysis led to three patient-level barriers that hinder the management of RA based on disease activity in daily clinical practice: Most patients are unfamiliar with disease activity measures, many patients are not equipped to be involved in treatment (decisions), and patients generally prefer not to change their current medication regimen. The DAS-pass strategy consists of two components aimed to lower these barriers. The first component, decision supportive information for patients, consists of an informational leaflet and a patient held record. The second component is guidance by a specialized rheumatology nurse. In **Table 1**, the full content of the DAS-pass strategy is mapped out. In this table, it can be seen that each subject is based on a strategy objective that is, in turn, based on a barrier.

**Table 1.** Content of the DAS-pass strategy.

**Content**	**Strategy element objectives**	Targeted barrier^*^
**Informational leaflet**		
Meaning of abbreviation ‘DAS28’	Increase knowledge	1
Variables in calculation of DAS28	Increase knowledge	1
Cut off values disease activity (low/moderate/high)	Increase knowledge	1
Recommended frequency of DAS28 monitoring	Increase knowledge Empowerment	1, 2
Recommended cut-off points for medication intensification	Increase knowledge	1
Importance of medication adaptation for clinical outcomes	Increase knowledge Change medication beliefs	1, 3
Importance of patient communicating about DAS28 with rheumatologist and nurse	Empowerment	2
Role of rheumatologist in balancing medication and side effects and/or personal situation patients	Increase knowledge	1
Patients quotes about positive experiences with DAS28	Empowerment	2
Websites on RA and on DAS28 (additional information)	Increase knowledge Empowerment	1, 2
**Patient held record**		
General personal information	Empowerment	2
DAS28 variables, calculated DAS28 and medication, per visit (table)	Empowerment Change medication beliefs	2, 3
DAS28 over time (graph)	Empowerment Change medication beliefs	2, 3
Questions to rheumatologist or nurse, per visit	Empowerment	2
**Guidance by specialized nurse**		
Explain contents of leaflet, tailored to patients’ needs	Increase knowledge Empowerment	1, 2
Explain patient held record, tailored to patients’ needs	Increase knowledge Empowerment	1, 2
Fill in first data patient held record	Empowerment	2
Stimulate asking questions	Increase knowledge Empowerment	1, 2
Stimulate use decision supportive information	Empowerment	2

* 1: Patients are not aware of (the importance of) disease activity; 2: Patients are not involved in their treatment (decisions); 3: Patients prefer not to change medication

### Component I: Decision supportive information for patients

#### Informational leaflet: ‘What is my disease activity?’

The leaflet ‘What is my disease activity’ is designed to educate patients about disease activity and its importance for clinical outcomes. By providing RA patients with information on RA management based on disease activity, the informational leaflet aims to increase patients’ knowledge on the subject. Furthermore, it is designed to change patients’ medication beliefs by emphasizing the importance of a strict medication policy for good clinical outcomes (see appendix material for examples). In addition, patients are encouraged to ask for their disease activity and discuss their medication options with their rheumatologist or specialized rheumatology nurse. By encouraging patients to address their disease activity with their clinicians, the leaflet aims to empower patients to be able to manage their own treatment.^32^

#### Patient held record: ‘The DAS-passport’

The DAS-passport is a patient-held record, where patients can write down their own DAS28 score in a table and a graph to see changes in their disease activity over time, and information on their RA medication (changes) and on the topics they want to discuss with their professional during the next consultation (see **Appendix** for examples). By adding an interactive component to the decision supportive information, patients should feel more involved and become better self-managers. In addition, the DAS-passport aims to increase the uptake of information. This should lead to an increased understanding of the concept of disease activity and the importance of keeping it low.^27,35,36^

### Component II: Guidance by a specialised nurse

A specialised rheumatology nurse discusses the decision supportive information (component 1) during an individual consultation with each patient. A checklist was developed to navigate nurses in this consultation. The nurse aims to stimulate patients to communicate about their disease activity during visits, to individualise the decision supportive information to the patients’ needs and to give the patients the opportunity to ask questions or ask for additional support. Guidance by a specialised rheumatology nurse is an important component of the patient-centred innovation strategy because it empowers patients to be more involved in their treatment in multiple ways. This component aims to optimise the uptake of information, activate patients, and improve their self-efficacy.^34^

## DISCUSSION

The DAS-pass strategy is a self-management strategy that aims to improve RA care by focusing on patient-level barriers to optimal disease activity management. It was developed through a process of co-design with stakeholders and based on successful strategies from other chronic diseases. Until now, none of the innovation strategies on RA management based on disease activity focused on barriers at patient level, while we know that these barriers exist and that patient-centred strategies are effective in other disciplines.^27–29^ Despite efforts to implement management based on disease activity measures into RA daily practice, we learned that the lack of patient awareness about the principle hinders rheumatologists in adhering to it.^14–19^ Empowering and educating patients can improve communication between patients and physicians and enable patients to take the initiative. In addition, when patients take initiative in monitoring their own disease activity using the DAS-passport, rheumatologists might be urged to follow the principle. Hence, the DAS-pass strategy should result in improved RA daily clinical practice and, ultimately, in patients experiencing less functional problems and less joint damage. The absence of patient-centred strategies does not mean that no previous efforts have been made to provide patients with education on disease management. With the growing attention for patient-centred care, patient education has become more important in RA management.^1,2^ However, with informational flyers alone, most patients do not have the proper tools. Patients need knowledge as well as empowerment to be able to play an important role in their own healthcare.^32^ The DAS-pass strategy addresses patients’ needs as it was based on patient-level barriers. Moreover, rheumatology nurses play an important role in the DAS-pass strategy because they tailor the strategy to patients’ individual needs and circumstances. Rheumatology nurses also play an important role in the embedding of the DAS-pass strategy in daily clinical practice. Typically, tasks of the rheumatology nurse include providing patient education and facilitating self-management.^37^ Providing them with carefully developed tools like the DAS-pass strategy can increase the effectiveness of nurses’ efforts and ensures the feasibility of the strategy in daily clinical practice.

One of the ways to tailor the instrument to patients’ needs is to digitalise it. Modern healthcare approaches such as electronic patient portals provide us with the opportunity to make information even more accessible to patients. In our view, the DAS-pass materials lend themselves perfectly for such applications. We heard several patients indicating a preference for a digitalised tool, while others preferred the paper-based version. Moreover, the current, paper-based materials allow us to also reach patients who are not used to using digitalised information, to evaluate the effects of the strategy. In addition, we view the role of the rheumatology nurse, discussing the decision supportive information, as a crucial component of our strategy.

Even though the DAS-pass strategy was developed carefully, the strategy is not likely to fit the needs of every RA patient. Research shows that there are patient groups that are unwilling or incapable of playing an important role in their own disease management.^21^ We believe that there are patients for whom the DAS-passport is an unfit tool. However, patients’ needs do change over time. From the literature, we know that early RA patients generally have other needs than more ‘experienced’ RA patients.^38,39^ This asks for a certain awareness of health professionals regarding patient education. To make education a continuous and integral part of RA management, patients’ needs need to be assessed regularly and tools need to be available to fit changing needs. Our DAS-pass strategy has the ability to be tailored to meet those changing needs because of the important role of the specialised rheumatology nurse.

A possible limitation of our study is that the barrier analysis by van Hulst et al. dates from 2008–2010. It is possible that patient-level barriers have shifted since. Furthermore, van Hulst et al. limited their barrier study to the Netherlands.^5,14,18^ Therefore it cannot be confirmed that results are generalisable to other countries. However, more recent studies and studies from other countries than the Netherlands describe similar patient-level barriers to optimal disease management in terms of treatment outcomes.^15–17,19^ Also, during our preliminary work, we used (among other materials) information materials on the Disease Activity Score by the National Rheumatoid Arthritis Society (United Kingdom), which indicates that RA patients in other European countries also need education about disease activity.^40^

We performed a Randomised Controlled Trial (RCT) to evaluate the effects of the DAS-pass strategy. In the Rheumatology department of a regional hospital in the Netherlands, 200 patients took part in the ‘What is my disease activity?’ trial. Of these patients, 100 received the DAS-pass strategy, whilst the other 100 patients received usual care. The RCT will show whether the DAS-pass strategy successfully empowers patients to be more involved in their treatment (decisions), improves their knowledge about disease activity in general and the DAS28 in particular, and improves their beliefs about (changing) medication. The preliminary outcomes of the strategy are positive. Rheumatologists and rheumatology nurses report that they receive positive feedback from patients who received the strategy (**Table 2**).

**Table 2.** Example of first responses to the DAS-pass strategy.

“*Repeatedly patients brought their DAS-passport to a visit. They like to have insight into their disease activity and ask me questions about the DAS28” (rheumatologist)*
*“When disease activity gradually increases while tapering, a patient does not always feel it. Using the chart in the DAS-passport, I can explain why it might be necessary to (temporarily) stop tapering” (rheumatologist)*

The DAS-pass strategy is a promising self-management approach to improving RA care as it was developed systematically: based on patient-level barriers, in a process of co-design with stakeholders, and based on successful strategies from other chronic diseases. By empowering and educating RA patients, the DAS-pass strategy aims to improve care through a patient-centred strategy: to improve communication about disease activity between patients and rheumatologists, and to enable patients to take the initiative in the management of their disease. The DAS-pass strategy was found helpful by stakeholders. This example shows how an evidence based, feasible and efficient strategy can be designed to improve patient knowledge about a chronic disease and involvement in its treatment.

Our strategy can be used to improve RA daily clinical practice. Our systematic approach can be used to improve patient knowledge and self-management on other RA related topics. Also, it can be used to improve the management of other chronic conditions. We therefore provide a detailed description of our methodology to assist those interested in the development of an evidence based strategy for empowering and educating patients.

## Figures and Tables

**Figure 1.**
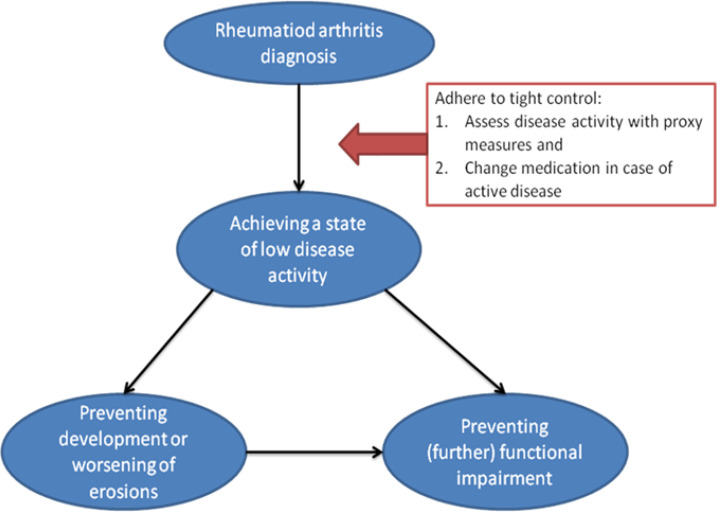
Informational leaflet; Importance of medication adaptation for clinical outcomes.

**Figure 2.**
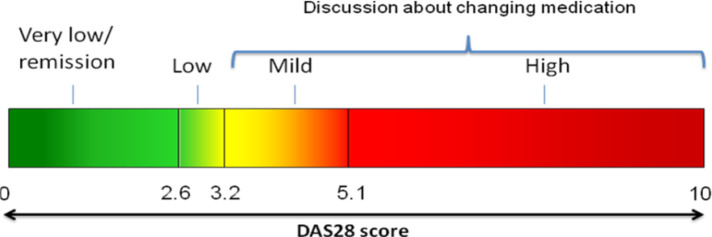
Informational leaflet; Recommended cut-off points for medication adaptation.

**Figure 3.**
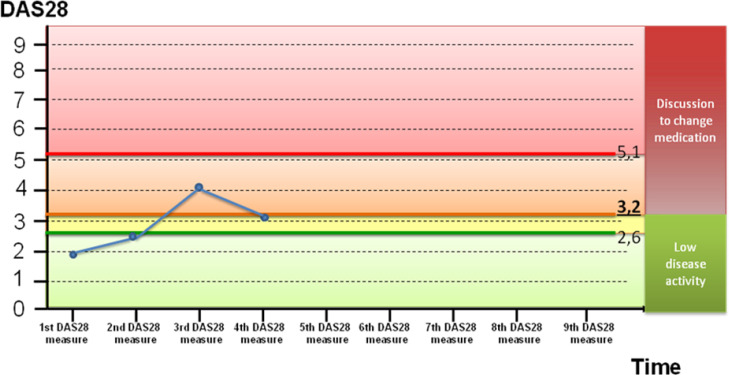
Patient held record; DAS28 over time (graph).

## ABBREVIATIONS

DAS: Disease Activity Score
DAS28: Disease Activity Score of 28 joints
IPDAS: International Patient Decision Aids Standards
RA: Rheumatoid Arthritis
RCT: Randomized Controlled Trial

## References

[1] Anderson J Caplan L Yazdany J Robbins ML Neogi T Michaud K Rheumatoid arthritis disease activity measures: American College of Rheumatology recommendations for use in clinical practice. Arthritis Care Res (Hoboken) 2012;64:640–7. 22473918 10.1002/acr.21649 PMC4028066

[2] Smolen JS Breedveld FC Burmester GR Bykerk V Dougados M Emery P Treating rheumatoid arthritis to target: 2014 update of the recommendations of an international task force. Ann Rheum Dis 2016;75:3–15. 25969430 10.1136/annrheumdis-2015-207524 PMC4717393

[3] van Riel PL. The development of the disease activity score (DAS) and the disease activity score using 28 joint counts (DAS28). Clin Exp Rheumatol 2014;32:S-65–74. 25365092

[4] Harrold LR Harrington JT Curtis JR Furst DE Bentley MJ Shan Y Prescribing practices in a US cohort of rheumatoid arthritis patients before and after publication of the American College of Rheumatology treatment recommendations. Arthritis Rheum 2012;64:630–8. 21953645 10.1002/art.33380 PMC3253907

[5] van Hulst LT Hulscher ME van Riel PL. Achieving tight control in rheumatoid arthritis. Rheumatology (Oxford) 2011;50:1729–31. 21933791 10.1093/rheumatology/ker325

[6] Yu Z Lu B Agosti J Bitton A Corrigan C Fraenkel L Implementation of treat-to-target for rheumatoid arthritis in the US: analysis of baseline data from a randomized controlled trial. Arthritis Care Res (Hoboken) 2018;70:801–6. 28834390 10.1002/acr.23343 PMC5823714

[7] Fransen J Moens HB Speyer I van Riel PL. Effectiveness of systematic monitoring of rheumatoid arthritis disease activity in daily practice: a multicentre, cluster randomised controlled trial. Ann Rheum Dis 2005;64:1294–8. 15829574 10.1136/ard.2004.030924 PMC1755664

[8] Goekoop-Ruiterman YP de Vries-Bouwstra JK Kerstens PJ Nielen MM Vos K van Schaardenburg D DAS-driven therapy versus routine care in patients with recent-onset active rheumatoid arthritis. Ann Rheum Dis 2010;69:65–9. 19155234 10.1136/ard.2008.097683

[9] Grigor C Capell H Stirling A McMahon AD Lock P Vallance R Effect of a treatment strategy of tight control for rheumatoid arthritis (the TICORA study): a single-blind randomised controlled trial. Lancet 2004;364:263–9. 15262104 10.1016/S0140-6736(04)16676-2

[10] Nikiphorou E Norton S Young A Carpenter L Dixey J Walsh DA Association between rheumatoid arthritis disease activity, progression of functional limitation and long-term risk of orthopaedic surgery: combined analysis of two prospective cohorts supports EULAR treat to target DAS thresholds. Ann Rheum Dis 2016;75:2080–6. 26979104 10.1136/annrheumdis-2015-208669 PMC5136699

[11] Schipper LG van Hulst LT Grol R van Riel PL Hulscher ME Fransen J. Meta-analysis of tight control strategies in rheumatoid arthritis: protocolized treatment has additional value with respect to the clinical outcome. Rheumatology (Oxford) 2010;49:2154–64. 20671022 10.1093/rheumatology/keq195

[12] Vermeer M Kuper HH Moens HJ Drossaers-Bakker KW van der Bijl AE van Riel PL Sustained beneficial effects of a protocolized treat-to-target strategy in very early rheumatoid arthritis: three-year results of the Dutch Rheumatoid Arthritis Monitoring remission induction cohort. Arthritis Care Res (Hoboken) 2013;65:1219–26. 23436821 10.1002/acr.21984

[13] Verstappen SM Jacobs JW van der Veen MJ Heurkens AH Schenk Y ter Borg EJ Intensive treatment with methotrexate in early rheumatoid arthritis: aiming for remission. Computer Assisted Management in Early Rheumatoid Arthritis (CAMERA, an open-label strategy trial). Ann Rheum Dis 2007;66:1443–9. 17519278 10.1136/ard.2007.071092 PMC2111604

[14] Van Hulst LT. Tight control in rheumatoid arthritis: bridging the gap between evidence and daily clinical practice. Nijmegen: Radboud University Medical Center 2010.

[15] Fraenkel L Cunningham M. High disease activity may not be sufficient to escalate care. Arthritis Care Res (Hoboken) 2014;66:197–203. 23926094 10.1002/acr.22098 PMC4072663

[16] Fraenkel L Seng EK Cunningham M Mattocks K. Understanding how patients (vs physicians) approach the decision to escalate treatment: a proposed conceptual model. Rheumatology (Oxford) 2015;54:278–85. 25172936 10.1093/rheumatology/keu324 PMC4301710

[17] Tymms K Zochling J Scott J Bird P Burnet S de Jager J Barriers to optimal disease control for rheumatoid arthritis patients with moderate and high disease activity. Arthritis Care Res (Hoboken) 2014;66:190–6. 23983001 10.1002/acr.22108

[18] van Hulst LT Kievit W van Bommel R van Riel PL Fraenkel L. Rheumatoid arthritis patients and rheumatologists approach the decision to escalate care differently: results of a maximum difference scaling experiment. Arthritis Care Res (Hoboken) 2011;63:1407–14. 21748861 10.1002/acr.20551 PMC3698485

[19] Wolfe F Michaud K. Resistance of rheumatoid arthritis patients to changing therapy: discordance between disease activity and patients’ treatment choices. Arthritis Rheum 2007;56:2135–42. 17599730 10.1002/art.22719

[20] Chilton F Collett RA. Treatment choices, preferences and decision-making by patients with rheumatoid arthritis. Musculoskeletal Care 2008;6:1–14. 17726671 10.1002/msc.110

[21] Nota I Drossaert CH Taal E van de Laar MA. Arthritis patients’ motives for (not) wanting to be involved in medical decision-making and the factors that hinder or promote patient involvement. Clin Rheumatol 2016;35:1225–35. 25392118 10.1007/s10067-014-2820-y

[22] Nota I Drossaert CH Taal E van de Laar MA. Patients’ considerations in the decision-making process of initiating disease-modifying antirheumatic drugs. Arthritis Care Res (Hoboken) 2015;67:956–64. 25504789 10.1002/acr.22531

[23] Nota I Drossaert CH Taal E Vonkeman HE van de Laar MA. Patient participation in decisions about disease modifying anti-rheumatic drugs: a cross-sectional survey. BMC Musculoskelet Disord 2014;15:333. 25281209 10.1186/1471-2474-15-333 PMC4192293

[24] Elwyn G Tilburt J Montori V. The ethical imperative for shared decision-making. Eur J Pers Cent Healthc 2013;1:129–31.

[25] Hudon C Fortin M Haggerty J Loignon C Lambert M Poitras ME. Patient-centered care in chronic disease management: a thematic analysis of the literature in family medicine. Patient Educ Couns 2012;88:170–6. 22360841 10.1016/j.pec.2012.01.009

[26] Dijkstra R Braspenning J Grol R. Empowering patients: how to implement a diabetes passport in hospital care. Patient Educ Couns 2002;47:173–7. 12191541 10.1016/s0738-3991(01)00196-3

[27] Grol R Grimshaw J. From best evidence to best practice: effective implementation of change in patients’ care. Lancet 2003;362:1225–30. 14568747 10.1016/S0140-6736(03)14546-1

[28] Duke SA Colagiuri S Colagiuri R. Individual patient education for people with type 2 diabetes mellitus. Cochrane Database Syst Rev 2009;1:CD005268. 10.1002/14651858.CD005268.pub2 PMC6486318 19160249

[29] van Peperstraten A Nelen W Grol R Zielhuis G Adang E Stalmeier P The effect of a multifaceted empowerment strategy on decision making about the number of embryos transferred in in vitro fertilisation: randomised controlled trial. BMJ 2010;341:c2501. 20884700 10.1136/bmj.c2501 PMC2948112

[30] Oshima Lee E Emanuel EJ. Shared decision making to improve care and reduce costs. N Engl J Med 2013;368:6–8. 23281971 10.1056/NEJMp1209500

[31] Stacey D Legare F Col NF Bennett CL Barry MJ Eden KB Decision aids for people facing health treatment or screening decisions. Cochrane Database Syst Rev 2017;4:CD001431. 28402085 10.1002/14651858.CD001431.pub5 PMC6478132

[32] Primdahl J Wagner L Holst R Horslev-Petersen K Group AS. The impact on self-efficacy of different types of follow-up care and disease status in patients with rheumatoid arthritis--a randomized trial. Patient Educ Couns 2012;88:121–8. 22386009 10.1016/j.pec.2012.01.012

[33] Elwyn G O’Connor A Stacey D Volk R Edwards A Coulter A Developing a quality criteria framework for patient decision aids: online international Delphi consensus process. BMJ 2006;333:417. 16908462 10.1136/bmj.38926.629329.AE PMC1553508

[34] Renders CM Valk GD Griffin S Wagner EH Eijk JT Assendelft WJ. Interventions to improve the management of diabetes mellitus in primary care, outpatient and community settings. Cochrane Database Syst Rev 2001;1:CD001481. 10.1002/14651858.CD001481 PMC7045779 11279717

[35] Dijkstra RF Braspenning JC Huijsmans Z Akkermans RP van Ballegooie E ten Have P Introduction of diabetes passports involving both patients and professionals to improve hospital out-patient diabetes care. Diabetes Res Clin Pract 2005;68:126–34. 15860240 10.1016/j.diabres.2004.09.020

[36] Holman H Lorig K. Patients as partners in managing chronic disease. Partnership is a prerequisite for effective and efficient health care. BMJ 2000;320:526–7. 10688539 10.1136/bmj.320.7234.526 PMC1117581

[37] Bech B Primdahl J van Tubergen A Voshaar M Zangi HA Barbosa L 2018 update of the EULAR recommendations for the role of the nurse in the management of chronic inflammatory arthritis. Ann Rheum Dis 2020;79:61–8. 31300458 10.1136/annrheumdis-2019-215458

[38] Makelainen P Vehvilainen-Julkunen K Pietila AM. Rheumatoid arthritis patients’ knowledge of the disease and its treatments: a descriptive study. Musculoskeletal Care 2009;7:31–44. 18697184 10.1002/msc.138

[39] Meyfroidt S Van der Elst K De Cock D Joly J Westhovens R Hulscher M Patient experiences with intensive combination-treatment strategies with glucocorticoids for early rheumatoid arthritis. Patient Educ Couns 2015;98:384–90. 25483574 10.1016/j.pec.2014.11.011

[40] Know your disease activity score (DAS) and stay one step ahead of RA. England and Wales: National Rheumatoid Arthritis Society 2015.

